# Return to Sport After Surgery for Adolescent Idiopathic Scoliosis: From Time-Based Clearance to Sport-Specific Readiness

**DOI:** 10.3390/jcm15166336

**Published:** 2026-08-16

**Authors:** Maria Cesarina May, Carlo Biz, Luca Puce, Andrea Zanirato, Pietro Ruggieri, Matteo Formica

**Affiliations:** 1 IRCCS Azienda Ospedaliera Metropolitana, 16132 Genoa, Italy; m_may_93@web.de (M.C.M.); andrea.zanirato@unige.it (A.Z.); matteo.formica@unige.it (M.F.); 2 Department of Orthopedics and Orthopedic Oncology, Department of Surgery, Oncology and Gastroenterology (DiSCOG), University of Padova, 35128 Padova, Italy; pietro.ruggieri@unipd.it; 3 Department of Neuroscience, Rehabilitation, Ophthalmology, Genetics, Maternal and Child Health (DINOGMI), University of Genoa, 16132 Genoa, Italy; 4 Department of Integrated Surgical Diagnostic Sciences (DISC), Orthopedic Clinic, University of Genoa, 16132 Genoa, Italy

**Keywords:** adolescent idiopathic scoliosis, return to sport, posterior spinal fusion, sports participation, rehabilitation, athletic performance, psychological readiness, vertebral body tethering

## Abstract

Return to sport (RTS) is an important outcome after surgery for adolescent idiopathic scoliosis (AIS), yet postoperative clearance remains largely time-based and reported outcomes vary according to procedure, population, endpoint, and treating-center policy. This critical review integrates direct postoperative RTS cohorts with biomechanical, rehabilitation, psychological, safety, and clinical-guidance evidence. Most available data concern adolescents treated with posterior spinal fusion and rely on self-reported participation. Resumption of physical activity or sport is common within the first postoperative year, but return to the same discipline, previous competitive level, and objectively verified performance is less consistently demonstrated. Evidence for anterior fusion is sparse, whereas vertebral body tethering is supported by smaller, selected cohorts and should be interpreted separately. Associations between RTS and distal fusion level or construct characteristics remain inconsistent, and biomechanical studies suggest redistribution rather than normalization of movement. Reported sport-related complications are uncommon, but existing cohorts are too small and lack exposure denominators to quantify rare events. No universal safe date or validated AIS-specific readiness battery currently exists. The proposed Multidimensional Scoliosis Return-to-Sport Framework therefore uses elapsed time as safety context while integrating clinical recovery, physical and psychological readiness, graded sport-specific exposure, and distinct return outcomes. The framework is evidence-informed and requires prospective validation.

## 1. Introduction

The success of surgery for adolescent idiopathic scoliosis (AIS) is no longer judged solely by radiographic correction and routine functional recovery. For adolescent athletes, the clinically relevant question is whether surgery permits return to training, competition, and previous performance. Although sport participation is generally possible after posterior spinal fusion, reported return rates and timing remain difficult to compare because populations, procedures, clearance policies, follow-up intervals, and definitions of return to sport (RTS) differ substantially [1,2,3,4,5,6,7,8,9,10,11].

RTS is not a single endpoint: resuming recreational physical activity, returning to the same sport, competing at the previous level, and recovering preoperative performance are distinct outcomes. Timing is also shaped by surgeon-imposed restrictions, so earlier return may reflect clearance policy and competitive opportunity as well as individual recovery [12,13,14,15].

Recent reviews have primarily summarized return rates, timing, and possible clinical predictors after posterior spinal fusion [9,10,11]. Accordingly, this critical review had two main aims: (1) to synthesize the evidence on RTS after surgery for AIS, including biomechanical consequences, reported return rates and timing, determinants of return, rehabilitation, psychological readiness, safety, and procedure-specific considerations; and (2) to develop an evidence-informed framework for RTS progression that moves beyond clearance based solely on postoperative time. Direct RTS evidence is dominated by posterior fusion; anterior fusion is discussed where relevant, and vertebral body tethering (VBT) is considered separately. The proposed Multidimensional Scoliosis Return-to-Sport Framework integrates clinical recovery, physical and psychological readiness, graded sport-specific exposure, and distinct return outcomes from participation to performance.

## 2. Literature Search and Evidence Synthesis

A targeted literature search was conducted in PubMed, Scopus, Web of Science, and Google Scholar and was last updated in May 2026. Reference lists of included articles and previous reviews were screened manually. Search terms combined “adolescent idiopathic scoliosis” with “spinal fusion”, “vertebral body tethering”, “return to sport”, “return to play”, “physical activity”, “athletic performance”, “biomechanics”, “rehabilitation”, and “psychological readiness”. Google Scholar was also used to identify relevant grey literature and professional guidance.

Original postoperative RTS studies constituted the primary evidence base. Biomechanical, rehabilitation, psychological, safety, and long-term studies, together with reviews, surveys, and consensus documents, were used to support clinical interpretation. Posterior fusion, anterior fusion, and VBT were considered separately when appropriate. Because populations, procedures, clearance policies, RTS definitions, and outcome measures were substantially heterogeneous, findings were synthesized narratively without quantitative pooling or formal risk-of-bias assessment.

## 3. Biomechanical Consequences of Spinal Fusion for Adolescent Idiopathic Scoliosis

**Figure 1:**
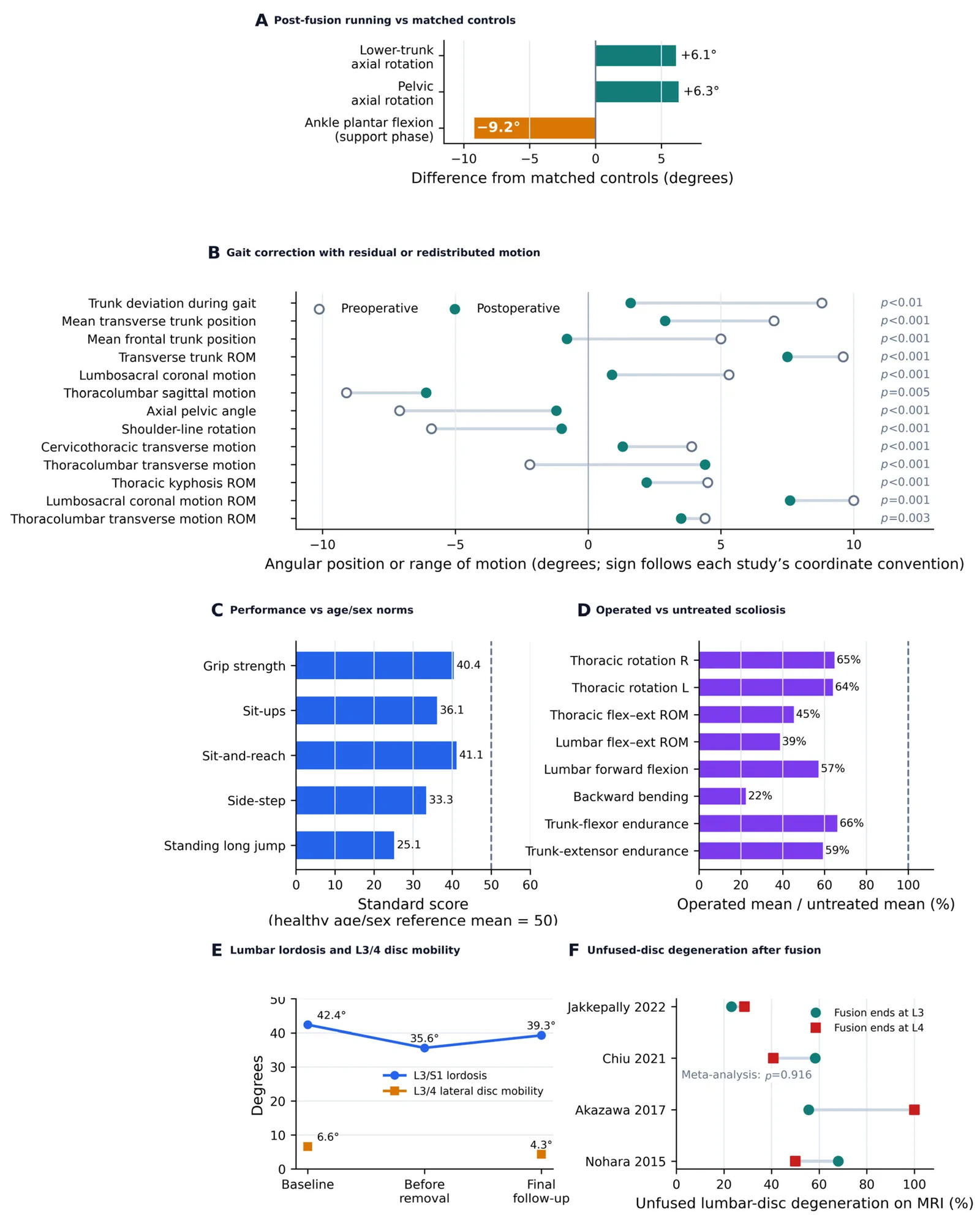
Selected biomechanical and functional findings after spinal fusion in AIS-relevant cohorts. (**A**) Running kinematics versus matched controls. (**B**) Pre- to postoperative gait kinematics and redistribution of motion. (**C**) Long-term physical performance relative to normative values. (**D**) Mobility and endurance in operated versus untreated idiopathic scoliosis. (**E**) Lordosis and L3–L4 mobility after temporary L4 fixation. (**F**) MRI-detected degeneration of unfused discs after fusion ending at L3 or L4. Values were extracted or calculated from published aggregate data; no pooling was performed. Sources: (A) [16]; (B) [17,18,19]; (C) [20]; (D) [21]; (E) [22]; (F) [23].

Spinal fusion creates a rigid instrumented block with residual mobile segments. Available evidence mainly describes kinematics, task performance, or radiographic proxies; direct data on internal loading are limited. Functional task completion therefore does not establish normalized mechanics (Figure 1).

### 3.1. Kinematic Redistribution During Walking and Running

Running can be maintained after fusion, but through redistributed motion. In ten operated patients compared with ten controls, lower-trunk and pelvic axial rotation were 6.1° and 6.3° greater, whereas ankle plantar flexion during stance was 9.2° lower [16]. A second study found broadly preserved global running coordination but reduced dissociation between the caudal trunk and pelvis [24], consistent with a more “en bloc” strategy.

Fusion may also reduce preoperative gait asymmetry. Trunk deviation during walking improved in eighteen patients [17], and a prospective cohort of 24 showed improved trunk position by three months but reduced transverse trunk range of motion [18]. Correction may therefore improve orientation and symmetry without restoring preoperative motion.

Residual spinal regions also appear to redistribute motion: thoracolumbar and lumbosacral sagittal motion were inversely related before surgery and during postoperative recovery [19]. These angular data, however, do not establish normalization of joint moments, muscle forces, or loading of the unfused segments.

### 3.2. Residual Mobility, Task Quality, and Physical Capacity

Postoperative range-of-motion restriction is most consistently observed in flexion and axial rotation [25]. Forward-roll testing in 36 patients showed a modest decline in technical quality at one year, greatest with longer and more caudal posterior fusion [26]. This suggests that task quality depends partly on residual mobile segments, although fusion length, approach, and curve type cannot be separated.

Long-term participation can coexist with measurable physical deficits. A small cohort assessed more than 27 years after fusion showed lower strength, flexibility, agility, and jump performance than national norms [20], but substantial participation bias limits causal inference. Historical studies also reported reduced lumbar mobility and trunk-extensor endurance [21], although these findings should not be extrapolated directly to modern constructs.

### 3.3. Distal Fusion Level, Adjacent Segments, and Long-Term Consequences

In selected lumbar curves, preserving one segment can retain measurable mobility. After transient L4 fixation and later screw removal, L3–L4 mobility remained about 65% of baseline and lordosis partially recovered [22]. This selected staged technique lacks randomized comparison with definitive fusion, so radiographic preservation of motion cannot be assumed to confer an athletic benefit.

Long-term sport participation remains heterogeneous. Historical cohorts also reported lower sport activity than controls, without a clear difference between surgically and nonsurgically treated patients [27]. At approximately 32 years of follow-up, 79.4% had resumed sport and 61.7% remained active, but only about half of sport participants had recovered a level similar to their preoperative level [28]. These data describe participation rather than sport-specific loading or performance.

Imaging evidence also does not show consistent protection from ending fusion at L3 rather than L4. Long-term cohorts and a meta-analysis of 14 studies (1264 patients) found no clear difference in disc degeneration between L3 and L4 termination, although clinical heterogeneity remained substantial [23,29]. The distal level is therefore biomechanically relevant but not a deterministic predictor of RTS or degeneration.

## 4. Current Evidence: RTS Is Not a Single Endpoint

RTS evidence after surgery for AIS is generally favorable, but reported endpoints range from unrestricted activity to recovery of the previous competitive level. These outcomes are not interchangeable and should not be combined into a single return rate.

### 4.1. Interpreting the Evidence: Endpoints, Denominators, and Clearance Policy

Direct RTS evidence consists mainly of small observational cohorts with heterogeneous populations, techniques, clearance policies, and self-reported outcomes. Reviews summarize achieved outcomes, whereas surgeon surveys and consensus statements primarily describe practice [9,10,11,12,13,14,15]. Because clearance policy imposes a protocol-dependent lower boundary on RTS, cohorts released at four to eight weeks cannot be compared directly with those restricted for six to twelve months. At minimum, studies should distinguish unrestricted physical activity, participation in any sport, return to the same sport, return to the previous competitive level, and objective recovery of performance, with explicit denominators. Table 1 summarizes the principal direct postoperative studies.

### 4.2. How Often Do Patients Return?

Most patients resume some form of sporting activity, but the proportion decreases as the endpoint becomes more selective. In the retrospective cohort by Fabricant et al., 25 of 42 previously athletic adolescents (59.5%) returned to an equal or higher activity level after a mean follow-up of 5.5 years [1]. Ruffilli et al. reported the same endpoint in 76 of 112 athletic patients (67.8%) after a mean of 50.3 months [4]. Returners were younger and had less severe curves, but the retrospective design cannot determine whether these variables independently influenced athletic recovery or instead reflected differences in selection, motivation, procedure, or sport demands.

More favorable results emerged from the later prospective Fabricant cohort: 93% of 106 patients resumed sport, and most returned to the same or a higher competitive level [6]. Distal fusion level was not associated with return to the same level, preoperative sport, or sport type. Pepke et al. further illustrated the importance of endpoint definition: sport participation increased from 78% before surgery to 89% after surgery, but only 33 of 113 patients (29.2%) resumed exactly the same discipline [7]. High overall participation can therefore coexist with substantial migration between sports.

In Khaleeq et al., 66 of 90 eligible patients completed the questionnaire; 63.6% reported return to contact sport and 91% perceived recovery of preoperative performance [8]. The latter estimate should not be interpreted as objectively verified recovery because of nonresponse, retrospective assessment, and the absence of functional testing. Overall, resumption of some sporting activity is common, whereas same-sport return, equivalent training volume, and restored competitive performance remain less certain.

### 4.3. When Do Patients Return?

Return times range from a few months to more than one year and depend on both the endpoint and the clearance policy. In the prospective cohort by Tarrant et al., 51.4% of 77 patients resumed unrestricted physical activity within 24 weeks and 88.5% within 52 weeks [2], but the endpoint did not require return to the same sport or competitive level. Sarwahi et al. distinguished daily activities, physical activities, and sport [3]. By three months, 43% had resumed running and 37% had returned to the gym; within six months, 54% of previous participants had resumed non-contact sport and 63% contact sport. Preoperative-level recovery was reported by 53% in non-contact sport and 79% in contact sport, although differing denominators prevent direct comparison. In Ruffilli’s cohort, clearance began at three months, yet most patients who recovered an equal or higher level returned later, showing that permission did not translate automatically into immediate RTS [4].

**Figure 2:**
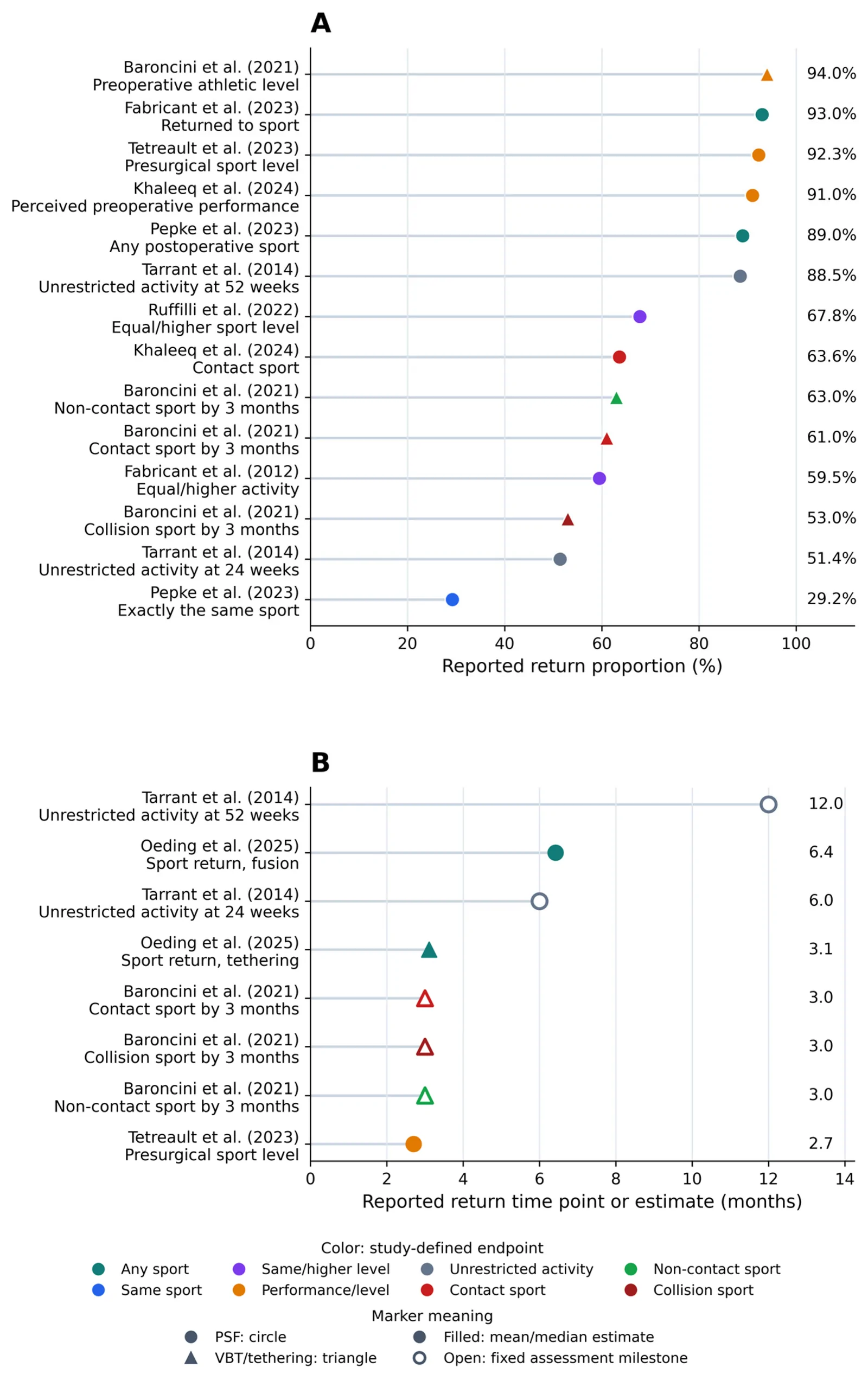
Study-defined return outcomes after surgery for AIS: reported proportions (**A**) and return-time estimates or assessment milestones (**B**). Color denotes the study-defined endpoint; circles represent fusion and triangles represent VBT. Filled markers indicate return estimates, whereas open markers indicate fixed assessment milestones. Sources: [1,2,4,5,6,7,8,30,31].

**Figure 3:**
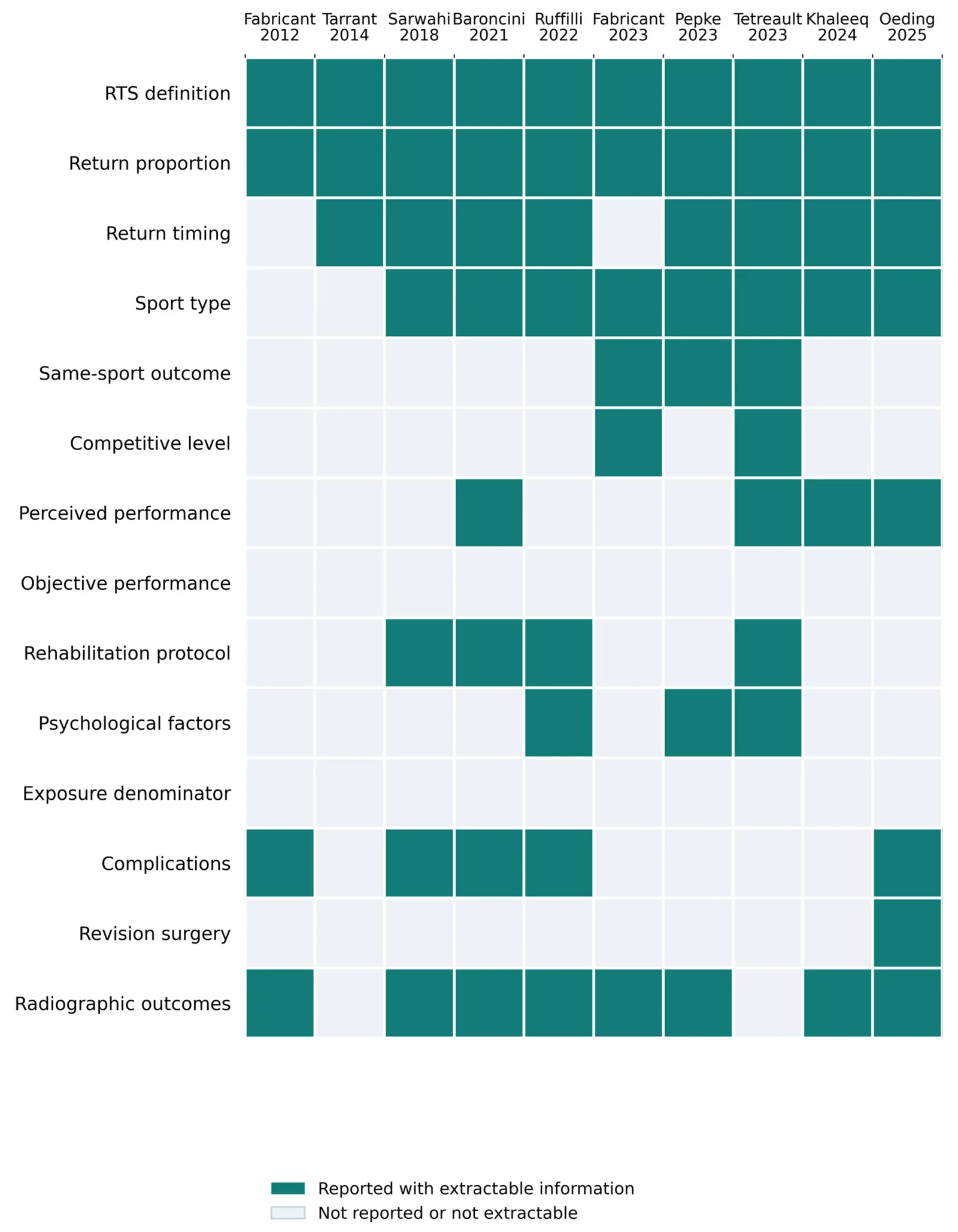
Reporting domains across the 10 direct postoperative RTS cohorts included in the review. Filled cells indicate extractable reporting; empty cells indicate absent or non-extractable information. Sources: [1,2,3,4,5,6,7,8,30,31].

Tetreault et al. studied 26 competitive athletes released for unrestricted activity at four to eight weeks; median patient-reported return to the presurgical level was 2.7 months, and 24 of 26 returned within one year [5]. Contact sport was slower to resume, while deconditioning and loss of flexibility were common barriers. The selected cohort and unusually early release policy limit generalizability. Across reviews, most reported returns occur within the first postoperative year, often between eight and twelve months, but this should not be interpreted as a biological threshold [11]. Figure 2 and Figure 3 summarize reported return outcomes and reporting domains.

### 4.4. Sport Type, Sport Migration, and Performance Recovery

The non-contact, contact, and collision classification is clinically convenient but biomechanically crude because it does not capture rotation, extreme flexion or extension, impact, fall risk, repetition, or fatigue. In Ruffilli’s cohort, ballet participation decreased from 44 patients before surgery to 4 at follow-up, whereas gym participation increased from 32 to 60 [4]. Among patients who did not recover their previous level, reported reasons included stiffness, advice from parents or clinicians, and fear of injury. Sport migration therefore reflects biomechanical, psychological, and contextual influences rather than mobility loss alone.

Pepke et al. also observed migration from contact toward non-contact activities, without a significant association between fusion length or distal level and time to return [7]. Khaleeq et al. reported mean return times of approximately seven to nine months in contact sport, but small Lenke subgroups preclude firm conclusions regarding curve type [8]. Changing sport is not necessarily surgical failure, while resuming the same sport does not by itself demonstrate restored technique, load tolerance, or performance.

### 4.5. Vertebral Body Tethering: A Separate Motion-Preserving Evidence Stream

VBT is a growth-modulating, motion-preserving procedure rather than a fusion technique. In a selected series of 31 patients, 94% reported return to their preoperative athletic level and more than half resumed non-contact, contact, and collision sports within three months [30]. A very small nonrandomized comparison also suggested earlier sport return after tethering than after posterior fusion, with similar final return rates [31]. These findings are vulnerable to selection and confounding by indication, and any early functional advantage must be balanced against tether failure, revision, and uncertain long-term durability.

### 4.6. The Safety Signal

Sport-related complications are rarely reported in RTS cohorts, and several studies observed no loss of correction, implant failure, or event attributed to sport [1,3,4]. However, absence of events in small cohorts cannot establish zero risk; for example, no events among 112 patients remains compatible with an approximate 95% upper risk bound of 2.7%. Because training exposure is rarely reported, reliable safety estimates require active surveillance and exposure-adjusted denominators [32,33,34].

## 5. Determinants of Return to Sport

Age, deformity, fusion extent, physical capacity, psychological factors, and sport demands have all been associated with RTS, but the evidence is observational and these variables are strongly interdependent.

### 5.1. Patient, Deformity, and Preoperative Sport

Age and deformity severity are among the most frequently reported correlates of RTS, although findings are inconsistent. In Ruffilli’s cohort, patients who returned to the same or a higher level were younger and had smaller main curves than non-returners [4]; among returners, younger age and lower curve magnitude were also associated with RTS within six months. A recent synthesis identified similar possible associations with later return [11]. These findings do not establish direct effects on athletic capacity. Greater curve severity may be linked to more extensive surgery and longer recovery, while age may track school transitions, changing sport interests, and psychological maturity. Age and Cobb angle may therefore be markers of a more complex pathway rather than independent causes of non-return.

Curve type has also been associated with RTS in some series [1,4,8], but interpretation is limited by collinearity among Lenke classification, deformity magnitude and stiffness, fusion length, and distal-level selection. In Khaleeq’s cohort, nonresponse and very small Lenke subgroups further reduced precision [8]. Preoperative athletic status introduces additional selection: the rapid-return cohort included motivated competitive athletes willing to complete monthly follow-up [5]. Future studies should record intention to return, discipline, competitive level, years of practice, and training volume rather than treating all preoperative activity as equivalent.

### 5.2. Fusion Extent, Surgical Approach, and Construct Characteristics

The lowest instrumented vertebra (LIV) is the most frequently studied anatomical determinant. Earlier cohorts suggested lower or later return with more distal fusion [1,3,4], although associations differed according to whether the endpoint was physical activity, early return, or recovery of the previous sport level.

More recent studies have not confirmed a consistent relationship. Pepke et al. found no association between the number of fused vertebrae or LIV and time to RTS [7], and the prospective Fabricant cohort found no association between LIV and return to the same competitive level, preoperative sport, or sport type [6]. Discordant findings likely reflect confounding by indication, collinearity between LIV and fusion length, limited measurement of residual mobility, and small numbers with distal lumbar fusion. A null association with general participation does not establish biomechanical irrelevance, particularly in sports requiring marked flexion or rotation. Inconsistent reporting of other construct characteristics, including screw density, construct rigidity, sagittal alignment, and surgical era, may further contribute to the discrepant findings across LIV studies because these factors vary between cohorts and are rarely modeled simultaneously. Consequently, the independent contribution of LIV to RTS cannot be reliably separated from the broader characteristics of the surgical construct.

Surgical approach is similarly underreported. Most direct RTS cohorts concern posterior fusion. In selected curves, anterior fusion may preserve more vertebral levels but includes discectomy and interbody arthrodesis, while thoracic or thoracoabdominal exposure introduces approach-specific morbidity that may affect early recovery [26,35]. Available studies cannot separate approach from curve type, fusion length, surgical era, and rehabilitation policy. Mobile segments should be preserved when compatible with stable, balanced correction, but no LIV, construct, or approach can currently be promised to improve RTS or performance.

### 5.3. Physical Capacity and Rehabilitation

Conditioning, flexibility, stiffness, and pain recur as barriers to return [4,5], yet rehabilitation is poorly described in direct RTS cohorts. Small postoperative rehabilitation studies suggest that physical capacity is modifiable, but they do not establish an RTS pathway [36,37]. Current consensus supports staged multidisciplinary recovery while leaving sport-specific milestones and objective readiness criteria largely undefined [15]. Prospective studies should therefore compare clearly described rehabilitation pathways using objective capacity, participation, performance, adherence, and exposure-adjusted safety outcomes.

### 5.4. Psychological and Social Context

Psychological recovery is heterogeneous. Catastrophizing and psychological flexibility have been associated with pain, function, quality of life, and activity recovery after AIS surgery [38,39], but these findings cannot be assumed to predict RTS. A small cognitive-behavioral study suggested modifiable body perception, although attrition was substantial and RTS was not assessed [40]. No AIS-specific psychological readiness scale for RTS has been validated; clinical assessment should therefore address confidence, fear, pain expectations, body image, motivation, and family support without unvalidated pass/fail thresholds.

## 6. From Time-Based Clearance to the MS-RTS Framework

The MS-RTS framework separates four tasks: define the intended outcome, characterize the demands of the athlete’s sport and position, distinguish medical clearance from performance restoration, and replace a single clearance event with monitored progression. It does not supersede restrictions required by infection, neurologic deficit, progressive pain, loss of correction, pseudarthrosis, or implant-related problems [32] and is intended to structure clinical reasoning rather than replace judgment.

### 6.1. Time Is a Necessary Context, but Not a Sufficient Criterion

Clinical practice has shifted toward earlier permission, but decisions remain predominantly calendar-based. Elapsed time provides context for wound healing, arthrodesis maturation, and construct stability, yet it does not directly measure fusion, capacity, or readiness. Surveys and consensus statements support gradual progression without a validated biological date or objective threshold [12,13,14,15]. A defensible model is therefore hybrid: time, clinical course, and radiographic findings establish a minimum safety context, while advancement depends on capacity, readiness, and tolerance of graded sport-specific exposure.

### 6.2. Stage 1: Clinical Recovery and Safety Context

Stage 1 determines whether functional progression is clinically appropriate. Core elements are a healed wound, controlled nonprogressive pain, no new neurologic or systemic findings, satisfactory alignment and instrumentation, and recovery of daily and school activities without relevant symptom exacerbation. Radiographs inform alignment and apparent construct integrity but do not directly measure biological maturity of the arthrodesis. Construct confidence therefore remains an integrated clinical judgment. New neurologic symptoms, increasing focal or night pain, systemic symptoms, loss of correction, or suspected implant problems require reassessment. Completion of Stage 1 permits more demanding capacity evaluation, not immediate competition.

### 6.3. Stage 2: Restoration of Foundational Physical Capacity

Stage 2 evaluates the athlete rather than the surgical result alone. Relevant domains include trunk and lower-limb strength and endurance, motor control, balance, aerobic capacity, movement quality, and repeated-load tolerance. Post-fusion tasks may be completed through compensatory strategies, so quality and fatigue response matter as much as task success [16,17,18,19,24]. Depending on age and sport, assessment may include trunk endurance, lower-limb strength and control, balance, running or landing tasks, submaximal aerobic capacity, and discipline-specific mobility. These measures can make clinical reasoning observable but do not constitute a validated MS-RTS battery. Preoperative values are preferable when available, appropriate norms are required, and symmetry may be misleading when both sides are deconditioned. No AIS-specific cutoff has been validated, and thresholds imported from other injuries should not be presented as evidence-based criteria for spinal fusion.

### 6.4. Stage 3: Graded Sport-Specific Exposure

Stage 3 transfers general capacity to sport through progressive exposure. Volume, intensity or speed, technical and decision-making complexity, and consequence of error should be increased separately when possible. Progression may begin with predictable individual drills before adding fatigue, rotation, impact, perturbation, and—where relevant—controlled then unpredictable contact. Assessment should include symptoms during and after the session, next-day recovery, movement quality under fatigue, confidence, and the ability to repeat the exposure. Pain escalation, neurologic symptoms, deteriorating control, or incomplete recovery should prompt regression. A single successful session demonstrates acute tolerance, not adaptation. This stage is particularly important in sports involving extreme mobility, repeated rotation, impact, falls, or collision and remains evidence-informed rather than prospectively validated [5,15].

### 6.5. Stage 4: Return to Participation, Sport, Competition, and Performance

Stage 4 distinguishes return to participation (modified training), return to sport (full practice), return to competition, and return to performance. These states may occur at different times, and athletes may move between them during recovery. Documentation should include training frequency and duration, completed sessions, remaining modifications, symptoms, recovery between exposures, athlete and coach feedback, and available technical or competitive metrics. Patient-reported performance is clinically meaningful but should be complemented, when feasible, by preoperative comparison, workload, technical measures, ranking, or competition data. A voluntary change in sport or level should be interpreted in context rather than automatically classified as success or failure.

### 6.6. Shared Decision Making and Framework Interpretation

Shared decision making is particularly important in collision, fall-risk, and elite sport, where potential consequences may be substantial and absolute risk remains uncertain. Current cohorts report few events but are underpowered and lack exposure denominators [1,3,4,5,6,7,8,9,10,11,32,33,34]. Discussion should address the available evidence, residual uncertainty, fusion characteristics, sport and position, competitive goals, risk tolerance, family perspective, and opportunities for modified progression. Surgeons, rehabilitation and sports professionals, coaches, athletes, and families should contribute to one coherent plan with defined responsibilities rather than parallel time-based restrictions.

**Figure 4:**
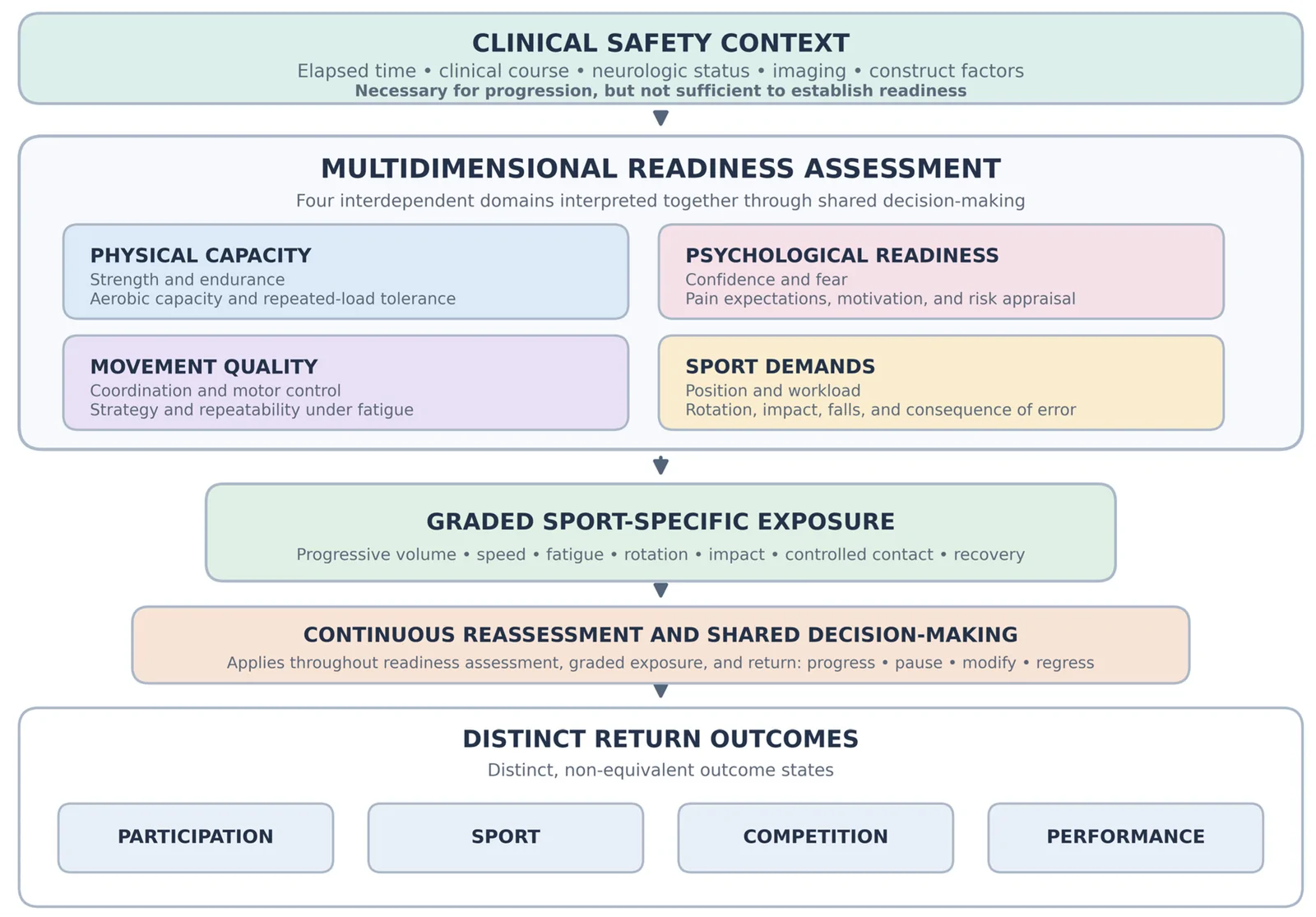
Proposed MS-RTS Framework integrating clinical safety context, multidimensional readiness, graded sport-specific exposure, continuous reassessment and shared decision-making, and distinct return outcomes. The framework is evidence-informed and has not yet been prospectively validated.

The Multidimensional Scoliosis Return-to-Sport Framework links clinical safety context, physical and psychological readiness, graded sport-specific exposure, and staged outcomes from participation to performance. It is an evidence-informed framework rather than a validated algorithm or clearance score. Figure 4 and Table 2 summarize its clinical implications. Prospective validation should establish feasibility, reliability, responsiveness, and association with meaningful performance, symptom, and safety outcomes.

## 7. Limitations of the Review

This critical review has limitations. The search was targeted rather than protocol-driven, and no formal risk-of-bias assessment or certainty grading was performed. The evidence base is small, heterogeneous, and dominated by single-center observational cohorts of female adolescents treated with posterior fusion and by self-reported endpoints. RTS definitions, denominators, follow-up, and clearance policies varied substantially, precluding quantitative pooling. Objective performance, rehabilitation exposure, training volume, and sport-related adverse events were rarely measured, and publication bias or overlap among institutional cohorts cannot be excluded. Historical, adult, and non-RTS studies were used only for interpretation. The proposed MS-RTS Framework has not been prospectively validated and should not be interpreted as a clearance algorithm or evidence for specific thresholds.

## 8. Future Directions

### 8.1. Define the Endpoint Before Testing Clearance

A core outcome set should distinguish participation, sport, competition, and performance and define the denominator for each [1,2,3,4,5,6,7,8,9,10,11]. Studies should record preoperative sport and intention to return, discipline and competitive level, training volume, procedure, rehabilitation and clearance policy, time from surgery and clearance, same-sport and same-level return, reasons for nonreturn or sport change, symptoms, performance, follow-up, and attrition. Reporting both surgery-to-RTS and clearance-to-RTS would help separate recovery from protocol-imposed restriction [12,13,14,15].

### 8.2. Test Pathways, Not Isolated Clearance Dates

Future studies should evaluate complete RTS pathways rather than isolated clearance dates. Prospective multicenter cohorts should begin before surgery and collect serial clinical, physical, psychological, and sport data through at least the first postoperative year. Criteria-informed pathways should be compared with usual time-based care, while safety should be reported per training hour or athlete-exposure [32,33,34]. Rehabilitation trials should describe intervention dose, progression, adherence, and professional expertise [36,37], and fusion–VBT comparisons should capture selection, correction, reoperation, and cumulative time away from sport [31].

### 8.3. Validate Readiness Measures and Targeted Technology

Candidate readiness measures should be validated before being combined into a score. Relevant domains include clinical and neurologic status, pain response, trunk and lower-limb capacity, balance and movement quality, aerobic fitness, sport-specific tasks, psychological readiness, and repeated-exposure tolerance. Validation should address reliability, responsiveness, construct validity, and prospective prediction of meaningful outcomes. Motion analysis, force platforms, electromyography, inertial sensors, and wearables may support longitudinal assessment, but their value depends on feasibility, interpretability, and whether they improve management beyond elapsed time, examination, and routine imaging [16,17,18,19,20,21,24,25,26,36,38,39,41].

### 8.4. Design Evidence for Underrepresented Patients, Procedures, and Sports

Current evidence is dominated by female adolescents treated with posterior fusion. Male, elite, adaptive, and collision-sport athletes, as well as sports involving falls, extreme mobility, repeated rotation, or high resistance, remain underrepresented. Evidence is also insufficient for anterior fusion, hybrid constructs, and other growth-modulating procedures. Future studies should describe sport mechanics explicitly and use prospective registries or longitudinal cohorts for rare populations rather than pooling clinically distinct scoliosis types.

## 9. Conclusions

Most adolescents undergoing contemporary posterior spinal fusion for AIS resume some form of physical activity or sport within the first postoperative year. Evidence is stronger for participation than for return to the same discipline, previous competitive level, objectively verified performance, or exposure-adjusted safety. Data for anterior fusion remain sparse, while VBT findings derive from small, selected cohorts and should be interpreted separately. Reported timing reflects endpoint definitions, population selection, surgical procedure, clearance policy, and opportunity to participate as well as recovery. Postoperative time is therefore necessary safety context but not a measure of readiness. The proposed MS-RTS Framework separates clinical safety from physical and psychological capacity, incorporates graded sport-specific exposure, and distinguishes participation, sport, competition, and performance. It should be viewed as an AIS-focused, evidence-informed structure for clinical reasoning that requires prospective multicenter validation, not as a validated cutoff or clearance score. Future research should also establish a standardized core outcome set that distinguishes return to participation, sport, competition, and performance and uses consistent definitions, denominators, and assessment time points across studies.

## Figures and Tables

**Table 1:** Direct postoperative evidence on return to sport after surgery for AIS.

Study	Design/Sample	Procedure and RTS Construct	Key Finding	Principal Limitation
Fabricant et al., 2012 [1]	Retrospective; *n* = 42 athletic adolescents	PSF; equal/higher preoperative activity	59.5% returned; distal fusion, Lenke type, and SRS-22 associated with return	Small single-surgeon cohort; retrospective endpoint
Tarrant et al., 2014 [2]	Prospective; *n* = 77	PSF; unrestricted physical activity	51.4% by 24 weeks; 88.5% by 52 weeks	Physical activity was not sport performance
Sarwahi et al., 2018 [3]	Retrospective survey; *n* = 95	PSF; validated SAQ categories	Substantial return to non-contact and contact sport by six months	Recall and surgeon-policy effects
Ruffilli et al., 2022 [4]	Retrospective; *n* = 112 athletic patients	High-density PSF; equal/higher sport level	67.8% returned; no RTS-related complications	Single-center, self-reported sport level
Tetreault et al., 2023 [5]	Prospective; *n* = 26 competitive athletes	PSF; presurgical sport and level	Median 2.7 months after 4–8-week release; 24/26 by one year	Highly selected small cohort; release policy embedded in outcome
Fabricant et al., 2023 [6]	Prospective; *n* = 106	PSF; same sport and competitive level	93% returned; no LIV association from T11-L4	Self-reported participation; rare-event safety not addressed
Pepke et al., 2023 [7]	Retrospective; *n* = 113	PSF; any sport and exactly the same sport	89% participated, but 29.2% resumed exactly the same sport	No nonsurgical control; maturation and preference confounding
Khaleeq et al., 2024 [8]	Retrospective questionnaire; 66/90 respondents	PSF; school, PE, running, sport, and perceived level	63.6% returned to contact sport; 91% perceived preoperative performance	Nonresponse, small Lenke subgroups, no objective performance
Baroncini et al., 2021 [30]	Retrospective questionnaire; *n* = 31	VBT; SAQ and preoperative athletic level	94% returned to preoperative level; early contact/collision return	No concurrent fusion control; selected VBT candidates
Oeding et al., 2025 [31]	Retrospective comparative; *n* = 22 for return outcomes	Combined tethering vs. PSIF	Sport return: 13.5 vs. 27.9 weeks	Very small nonrandomized groups; correction/revision tradeoffs

Abbreviations: LIV, lowest instrumented vertebra; PE, physical education; PSF, posterior spinal fusion; PSIF, posterior spinal instrumentation and fusion; RTS, return to sport; SAQ, Sports Activity Questionnaire; SRS-22, Scoliosis Research Society-22; VBT, vertebral body tethering.

**Table 2:** Evidence-to-practice synthesis of proposed MS-RTS domains and research priorities.

Domain	Current Evidence	Clinical Implication	Main Uncertainty/Research Priority
Endpoint definition	Participation is commonly reported; same-sport and performance recovery are less certain [1,2,3,4,5,6,7,8,11]	Document return to participation, sport, competition, and performance separately	Adopt a core outcome set with standardized definitions and time points
Timing and release	Observed timing partly reflects when return was permitted [2,5,12,13,14,15]	Use elapsed time as safety context, not as the sole readiness gate	Compare criterion-informed progression with usual care
Fusion extent/approach/construct	Associations with distal fusion are inconsistent, and approach-specific RTS evidence is sparse [1,3,4,6,7,26,35]	Preserve motion where surgically appropriate, without deterministic promises about level or approach	Develop prospective anatomy- and procedure-stratified cohorts using sport-relevant tasks
Sport demand and contact	Broad contact labels are crude, and rare events are poorly quantified [4,5,7]	Specify position, falls, rotation, collision frequency, and workload	Establish exposure-adjusted safety registries with active adverse-event surveillance
Physical capacity and rehabilitation	Conditioning, flexibility, and pain recur as barriers; rehabilitation is poorly described [2,4,5,7,15]	Use structured graded rehabilitation and monitor repeated-load tolerance	Test content, dose, adherence, and sport-specific pathways
Psychological readiness	Fear, confidence, family, and clinician beliefs influence recovery [38,39,40]	Assess readiness explicitly without an unvalidated pass/fail cutoff	Validate an AIS-specific psychological-readiness measure
VBT	Earlier return may occur in selected patients, but comparative data are limited [30,31]	Base procedure choice on the total clinical indication	Assess long-term comparative effectiveness, revision, and durability
Underrepresented populations	Male, elite, adaptive, collision, and flexibility-dependent athletes remain sparse	Avoid importing average AIS timelines into mechanically distinct settings	Develop purpose-built cohorts for underrepresented patients, procedures, and sports

AIS, adolescent idiopathic scoliosis; MS-RTS, Multidimensional Scoliosis Return-to-Sport; VBT, vertebral body tethering. This synthesis is not a validated clearance algorithm.

## Data Availability

No new primary data were generated. All study-level information summarized or derived in this review is available in the cited publications.
